# Emotional regulation in non-suicidal self-injury – research on the use of transcranial direct current stimulation (tDCS)

**DOI:** 10.1192/j.eurpsy.2022.1132

**Published:** 2022-09-01

**Authors:** I. Makowska, K. Rymarczyk, D. Puzio, K. Pałka-Szafraniec, J. Garnier

**Affiliations:** 1 Medical University of Lodz, Child And Adolescent Psychiatry Unit, Lodz, Poland; 2 SWPS University of Social Sciences and Humanities, Faculty Of Psychology, Warszawa, Poland

**Keywords:** emotion regulation, NSSI, TdCS, Adolescents

## Abstract

**Introduction:**

DSM-5 defines non-suicidal self-injury (NSSI) as socially unaccepted, direct, repeated and deliberate harm done to one’s own body. It is estimated that in a general population approximately 13-29% of adolescents present NSSI, and 70-80% among hospitalized youth. It seems that emotional dysregulation is the core characteristic of NSSI manifesting by self-harm behaviors, impulsiveness, lack of emotional awareness and experiencing high intensity of negative emotion. Emotional dysregulation is a pivotal characteristic of NSSI. Rationale of this theory is provided by the results of psychological and psychophysiological studies as well as those presenting brain activity. Neuroimaging data point to a variant pattern of brain activity of adolescents with NSSI during perception of emotionally negative stimuli i.e. hyperactivity in amygdala – a structure responsible for fear and automatic reaction to exciting stimuli and low activity of inferior frontal gyrus area – a structure responsible for inhibition and interpretation of social interactions. This activity pattern suggests a disorder of cortico-subcortical neuronal connections.

**Objectives:**

The aim was to verify tDCS as a therapeutic aid for patients who exhibit NSSI despite implementation of pharmacotherapy and psychotherapy.

**Methods:**

We investigated the modulation effect of tDCS treatment at the right inferior frontal gyrus (rIFG) in hospitalized adolescents with NSSI.

**Results:**

Preliminary tDCS stimulation results indicate potential usefulness of this method in regulating emotions and improving executive functions.

**Conclusions:**

Prefrontal cortex stimulation may restore balance in aforementioned connections and, as a result, positively influence an emotional regulation i.e. lower the impulsiveness, agitation and, by doing so, decrease NSSI frequency.

**Disclosure:**

No significant relationships.

